# Discussing the Prognosis and Complications of Transvenous Lead Extraction in Patients With Cardiac Implantable Electronic Devices (CIED): A Systematic Review

**DOI:** 10.7759/cureus.45048

**Published:** 2023-09-11

**Authors:** Korlos Salib, Lana Dardari, Maher Taha, Purva Dahat, Stacy Toriola, Travis Satnarine, Zareen Zohara, Ademiniyi Adelekun, Kofi D Seffah, Safeera Khan

**Affiliations:** 1 Internal Medicine, California Institute of Behavioral Neurosciences & Psychology, Fairfield, USA; 2 Pathology, California Institute of Behavioral Neurosciences & Psychology, Fairfield, USA; 3 Pediatrics, California Institute of Behavioral Neurosciences & Psychology, Fairfield, USA; 4 Family Medicine, California Institute of Behavioral Neurosciences & Psychology, Fairfield, USA; 5 Internal Medicine, Piedmont Athens Regional Medical, Athens, GRC

**Keywords:** complications, prognosis, transvenous lead extraction, defibrillator, implantable pacemakers, cardiac implantable electronic devices

## Abstract

An increase in cardiovascular implantable electronic devices (CIEDs) and undoubtedly the complications brought on by these devices coincide with an increase in cardiovascular disorders, particularly heart rhythm abnormalities. The safest procedure to extract these devices is transvenous lead extraction (TLE). Thus, this systematic review aimed to summarize the possibility of success rates and the common complications that could arise during the surgery. Full-text publications in PubMed, MEDLINE, PubMed Central (PMC), and ScienceDirect were used in this study, which was conducted using the Preferred Reporting Items for Systematic Reviews and Meta-Analyses (PRISMA) guidelines. Seventeen studies were reviewed for this systematic review after being screened by title, abstract, full-text availability, and quality appraisal assessment. Heart and vascular tears, along with tricuspid regurgitation (TR), are common adverse events. Pulmonary embolism, hemothorax, hemopericardium, and ghost appearance in echo are less common consequences. In addition, the longer the dwelling time of the leads, the greater the chance of infection due to an increase in lead adhesions and fibrous tissue that has made the procedure unsafe as time passes. However, we concluded that TLE is a successful method across all age groups with an excellent probability of clinical and procedural success in a majority of studies.

## Introduction and background

It is estimated that there are currently 3.5 million cardiac implantable electronic devices (CIED) implanted worldwide, and between 500,000 and 1,000,000 new leads are implanted yearly [1,2]. ​​CIEDs decrease the prevalence of mortality and morbidity in patients with cardiovascular diseases, such as bradycardias, heart failure, and ventricular arrhythmias [3,4]. The more implantable CIEDs there seems to be, the more complications we encounter. The prevalence of CIEDs is rising globally, and problems, such as infection, venous stenosis, or lead malfunction, are more likely to occur [3].

The cornerstone of the management of infected CIEDs and malfunctioning leads is transvenous lead extraction (TLE) [5]. In 1980, the first operational cases of lead extraction by continuous traction were carried out [6]. In the early 1990s, a different intravascular approach for CIED lead extraction was used along with the outer sheaths to break down fibrotic adhesion and extract the lead [7]. TLE has undergone many advances since the start of the 21st century, including the use of rotating mechanical dilator sheaths and ablative energy sources, such as excimer laser sheaths. Despite the development of new techniques, many of these interventions may have adverse outcomes, such as lead infection and lead failure [8-10].

Because of its excellent success rates and low risk of complications, percutaneous TLE is now favored over surgical lead extraction [4]. However, there is a slight chance of serious consequences, such as cardiac avulsion, vascular rupture, and death, with percutaneous TLE [4]. Therefore, TLE is an extremely difficult procedure. An experienced surgical team, including an electrophysiologist, cardiac surgeon, anesthesiologist, and cardiac radiologist, is necessary for a safe and effective procedure [11].

Several risk factors, including chronic kidney disease (CKD), chronic heart failure (CHF), anemia, and significant weight loss, can raise the chance of CIED extraction complications [12]. In addition, it has been observed that removing implantable defibrillators can result in more complications than any other types of CIEDs [13]. It is also crucial to mention that the clinical and procedural success rates of TLE in elderly people (>65 years old) are approximately the same as those in non-elderly people (<65 years old). It concludes that being elderly is not a risk factor for TLE problems [14]. Considerable intravascular lead adhesion, calcium buildup, and vascular occlusions may be caused by many leads and their prolonged dwell time, which raises the perceived risk of complications [15].

The costs and burdens of CIED infection only, without including other indications of TLE, are now the focus of much research, and it is estimated to range between $16,651 to $362,606 in the USA and €36,931 in the UK [16]. The mean cost for TLE is £10,727, and when device reimplantation is considered as well, the cost rises to £22,615 [17]. These massive costs motivated us to conduct this systematic review study and discuss the prognosis and the most common and potentially fatal complications of the procedure so that doctors can anticipate the conditions they may encounter and try to avoid them if at all possible.

## Review

Methods

This systematic review was written according to the Preferred Reporting Items for Systematic Reviews and Meta-Analyses (PRISMA) 2020 guidelines [18]. Keywords, including "cardiac implantable electronic devices," "implantable pacemakers," "defibrillator," "transvenous lead extraction," "prognosis," and "complications," were used. 

The literature search was performed on February 2023. We conducted our search using the following databases: PubMed, MEDLINE, PubMed Central (PMC), PubMed Medical Subject Heading (MeSH), and ScienceDirect. "Cardiac implantable electronic devices (CIED)," "transvenous Lead Extraction (TLE)," "prognosis," and "complications" were the search terms selected. Furthermore, the following were included in the PubMed MeSH search (Table 1):

**Table 1:** PubMed Medical Subject Heading (MeSH) strategies

(((( "Defibrillators, Implantable/adverse effects"[Mesh] OR "Defibrillators, Implantable/microbiology"[Mesh] )) OR ( "Pacemaker, Artificial/adverse effects"[Mesh] OR "Pacemaker, Artificial/microbiology"[Mesh] )) OR ( "Cardiac Resynchronization Therapy Devices/adverse effects"[Mesh] OR "Cardiac Resynchronization Therapy Devices/microbiology"[Mesh] ) OR "artificial pacemakers" OR "Cardiac Resynchronization Therapy Devices" OR "Implantable Cardioverter Defibrillators" OR ICD OR "cardiac implantable electronic devices" OR CIED AND ((y_5[Filter]) AND (humans[Filter]) AND (english[Filter]))) AND ("Transvenous lead extraction" OR "lead removal" OR TLE AND ((y_5[Filter]) AND (humans[Filter]) AND (english[Filter])))) AND ("Prognosis"[Majr] OR Prognosis AND ((y_5[Filter]) AND (humans[Filter]) AND (english[Filter])))	112
((((( "Defibrillators, Implantable/adverse effects"[Mesh] OR "Defibrillators, Implantable/microbiology"[Mesh] )) OR ( "Pacemaker, Artificial/adverse effects"[Mesh] OR "Pacemaker, Artificial/microbiology"[Mesh] )) OR ( "Cardiac Resynchronization Therapy Devices/adverse effects"[Mesh] OR "Cardiac Resynchronization Therapy Devices/microbiology"[Mesh] ) OR "artificial pacemakers" OR "Cardiac Resynchronization Therapy Devices" OR "Implantable Cardioverter Defibrillators" OR ICD OR "cardiac implantable electronic devices" OR CIED AND ((y_5[Filter]) AND (humans[Filter]) AND (english[Filter]))) AND ("Transvenous lead extraction" OR "lead removal" OR TLE AND ((y_5[Filter]) AND (humans[Filter]) AND (english[Filter])))) AND ("complications" [Subheading] OR complications OR "Adverse events" OR "Side effects" AND ((y_5[Filter]) AND (humans[Filter]) AND (english[Filter])))	159

**Figure 1:**
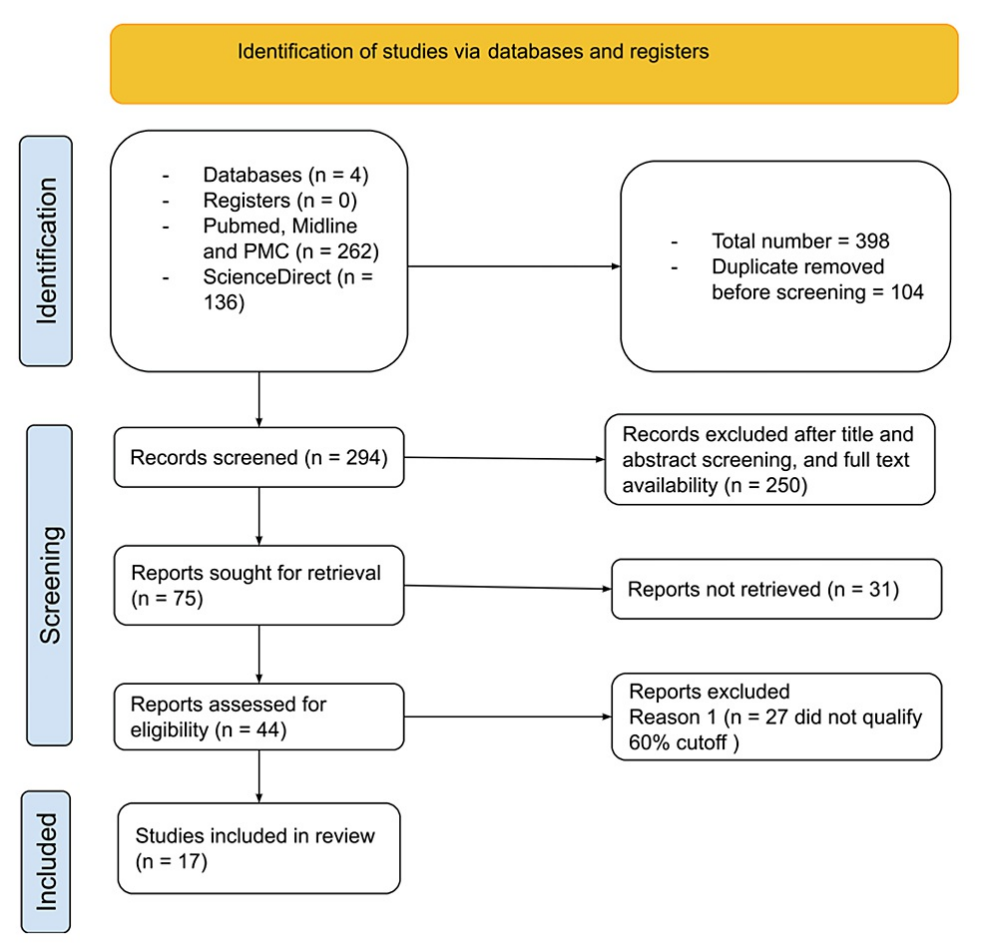
Identification of studies via databases and registers

After collecting publications that met the inclusion and exclusion criteria, the authors further focused their selection depending on the requirements of the study (Table 2). Manual duplicate detection and deletion were done. At this stage, abstracts, titles, and full-text availability were reviewed. The chosen candidates were then evaluated for quality (Figure 1).

**Table 2:** Inclusion and exclusion criteria

Inclusion criteria	Exclusion criteria
Papers written and published in English language or translated from another language into English	Gray literature
Papers focussing on patients of all age groups	Papers published before 2018
Papers focusing on humans only	Opinion texts
Papers discussing the three types of CIEDs: pacemakers, defibrillators, and cardiac resynchronization therapy	Papers discussing leadless pacemakers
Full text articles	

Results

Article selection, evaluation, and analysis were carried out at each stage. The team's decision was used to review an article's full text if there were any discrepancies in its findings about its suitability for inclusion. AXIS Scale was used to evaluate the cross-sectional studies (Table 3). The Newcastle-Ottawa Scale was used to assess observational studies, including case-control and cohort studies (Tables 4, 5, 6). The Joanna Briggs Institute (JBI) checklist was implemented to evaluate case-report studies (Table 7).

**Table 3:** Three cross-sectional studies were selected after the quality appraisal phase

	Monsefi et al. (2019) [3]	Mkoko et al. (2021) [4]	Tułecki et al. (2021) [19]
1. Are the aims/objectives of the study clear?	Yes	Yes	Yes
2. Was the study design appropriate for the stated aim?	Yes	Yes	Yes
3. Was the sample size justified?	Yes	No	Yes
4. Was the target/reference population clearly defined?	Yes	Yes	Yes
5. Was the sample frame taken from an appropriate population base so that it closely represented the target/reference population under investigation?	Yes	Yes	Yes
6. Was the selection process likely to select subjects/participants that were representative of the target/reference population under investigation?	Yes	Yes	Yes
7. Were measures undertaken to address and categorize non-responders?	No	No	No
8. Were the risk factor and outcome variables measured appropriate to the aims of the study?	Yes	Yes	Yes
9. Were the risk factor and outcome variables measured correctly using instruments/measurements that had been trialed, piloted, or published previously?	Yes	Yes	Yes
10. Is it clear what was used to determine statistical significance and/or precision estimates?	No	Limited	Yes
11. Were the methods (including statistical methods) sufficiently described to enable them to be repeated?	No	Limited	Yes
12. Were the basic data adequately described?	Yes	Yes	No
13. Does the response rate raise concerns about non-response bias?	No	No	No
14. If appropriate, was information about non-responders described?	No	No	No
15. Were the results internally consistent?	Yes	Yes	Yes
16 Were the results presented for all the analyses described in the methods?	Yes	Yes	Yes
17 Were the authors' discussions and conclusions justified by the results?	Yes	Yes	Yes
18 Were the limitations of the study discussed?	No	No	Yes
19 Were there any funding sources or conflicts of interest that may affect the authors’ interpretation of the results?	Yes	No	Yes
20 Was ethical approval or consent of participants attained?	Yes	Yes	Yes
Score	14/20	12/20	16/20

**Table 4:** Five cohort studies were selected after the quality appraisal phase

	Aleong et al. (2020) [13]	Chung et al. (2022) [20]	Burger et al. (2021) [21]	Zabak et al. (2023) [22]	Segreti et al. (2019) [23]
Exposed people defined	Yes	Yes	Yes	Yes	Yes
Non-exposed people defined	No	No	No	No	No
Was the exposure and outcome clearly explained?	Yes	Yes	Yes	Yes	Yes
Was the outcome of interest present at the start of the study?	Yes	Yes	Yes	Yes	Unclear
Were the groups similar?	No	No	No	Yes	Unclear
The exposure and the outcomes measured the same way in both groups	No	No	No	Yes	Yes
Follow-up done correctly	Yes	Yes	Yes	Yes	Yes
Is the study published in an indexed journal?	Yes	Yes	Yes	Yes	Yes
Score	5/8	5/8	5/8	7/8	5/8

**Table 5:** Two cases of case-control studies were selected after the quality appraisal phase

	Zhou et al. (2021) [14]	Zabak et al. (2020) [24]
Case defined properly	Yes	Yes
Control defined properly	Yes	Yes
Exposure and outcome defined properly	Yes	Yes
Similarities between the groups	No	No
Chances of the groups being exposed to the exposure	Yes	Yes
The exposure and outcomes measured the same way in both groups	Yes	Yes
Follow-up done correctly	Yes	No
Is the study published in an indexed journal?	Yes	Yes
Score	7/8	6/8

**Table 6:** Four cases of observational studies were selected after the quality appraisal phase

	Representative of the population	Exposure of interest clearly defined	Comparison group clearly defined	Selection of the non-exposed	Confounding factors adjusted for in the analysis	Outcome of interest clearly defined	Follow-up periods similar across all participants	Ascertainment of exposure	Outcome was not known at the start of the study	Published in an indexed journal	Score
Park et al. (2018) [25]	Yes	Yes	No	No	No	Yes	Yes	Yes	No	Yes	6/10
Polewczyk et al. [26]	Yes	Yes	No	No	Yes	Yes	Yes	Yes	Unclear	Yes	7/10
Nowosielecka et al. (2022) [27]	Yes	Yes	No	No	Yes	Yes	Yes	Yes	No	Yes	7/10
Pecha et al. (2021) [28]	Yes	Yes	Yes	Yes	No	Yes	Yes	Yes	No	Yes	8/10

**Table 7:** Three cases of case report studies were selected after the quality appraisal phase

	Hayashi et al. (2022) [29]	Dunn et al. (2022) [30]	El- zein et al. (2020) [31]
Patients demography	No	No	No
Patients’ history clearly defined	Yes	Yes	Yes
Current clinical condition clearly described	Yes	Yes	Yes
Diagnostic tests results clearly described	Yes	Yes	Yes
Intervention/treatment clearly described	No	Yes	No
Post-intervention clinical condition clearly described	Yes	Yes	Yes
Adverse events identified	Yes	Yes	Yes
Does the case report have a takeaway message?	Yes	Yes	Yes
Is the study ethically approved?	Yes	Yes	Yes
Score	7/9	8/9	7/9

Discussion

Clinical success was defined as the accomplishment of all clinical targets related to the rationale for TLE or the retention of a little portion of lead (less than 0.4 cm) that has no detrimental effects on the procedure's intended results with the absence of any major complications [3,4]. Clinical failure was defined as a major procedure-related complication or as failing to achieve the intended clinical outcome of TLE [3,4]. Complete procedural success is referred to as extracting all targeted leads and all lead materials from the vascular space without any permanent debilitating complications or mortality [3]. Clinical success rates in the majority of the above studies, which exceed 90%, demonstrate that the procedure is generally safe [3,4,13,19,20].

Several risk factors may increase the risk of CIED extraction complications, such as CKD, CHF, anemia, and significant weight loss [20]. In-hospital mortality was more than two times more likely in patients with CKD, 3.6 times more likely in patients with CHF, and 3.8 times more likely in patients with previous records of significant weight loss [20].

As seen in studies number three, four, and 20, infection, whether local or systemic, is the most common indication of the procedure (Table 8). Study number 13 revealed, by contrast, that 59.8% of the patients have undergone TLE due to non-infectious causes [13]. *Staphylococcus aureus* is the most frequent organism of device-related infection bacterium [13]. When infected leads were extracted by TLE, there was a significant risk of mortality and morbidity if *S. aureus* was the source of infection. According to Aleong et al., *S. aureus* infections were the main cause of death in 40 patients and 149 serious complications [13]. 

**Table 8:** Prevalence of indications, success, and complication rates of TLE

	Year of publication	Number of patients with leads extracted	Number of leads extracted	Number of patients with infection indication / %	Number of patients with non-infection indication / %	Clinical success rate	Procedure success rate	Major complication number / (%)	Minor complication number / (%)
Tułecki et al. (2021) [19]	2021	1500		230 (15.33%)	1,270 (84.6%)	98.93%	96.13%	33 (2.20%)	115 (7.67%)
Monsefi et al. (2019) [3]	2018	108	277	79 (73.1%)	27 (26.9%)	98%	98.7%	15	
Mkoko et al. (2021) [4]	2021	53	75	69.2%	30.8%	96.2%	84.6%	1 (1.9%)	1(1.9%)
Aleong et al. (2020) [13]	2020	8304	57220	9,196 (16.1%)	34,240 (59.8%)			3,298 (5.8%)	
Chung et al. (2022) [20]	2022	510	1181	387 (75.9%)	123 (24.1%)	496 (97.1%)	471 (92.4%)	9 (1.80%)	9 (1.80%)

There have been reports of minor complications, such as pericardial effusion, blood transfusion, surgical site hematoma, and pneumothorax [19]. Major consequences include hemopericardium, hemothorax, severe TV damage, and vascular and cardiac tear and damage [19]. Tricuspid regurgitation (TR), which is considered minor if it diminishes valve function by two grades or less, is the most frequent minor complication of TLE according to study number 19. In addition, in the same study, hemopericardium is the major procedural consequence [19]. According to Chung et al., seven out of nine patients developed a pocket hematoma as a mild complication, and six out of nine patients experienced cardiac and vascular avulsion or tear [19]. 

According to study number three and 32, the 30-day mortality rates are 3.7% and 2.1%, respectively [3,32]. There is also a 1.9% hospital mortality rate in study number four [4]. In study number 32, the death rates are 8.4% and 46.8%, respectively, after one year and 10 years [32]. According to Chung et al., there are 30 patients with all-cause death or 5.9%, and septic shock is the leading cause of death, accounting for 60% of all fatalities, while procedure-related mortality comprised 1% of the total deaths in the same study [20]. 

Tricuspid Regurgitation

TR is one of the most prevalent TLE complications. Compared to implantable cardioverter defibrillators (ICDs), pacemaker lead extraction is more susceptible to causing TR [25,26]. Moreover, the extraction of two or more leads, the use of three or more extraction tools, and the indwelling duration of right ventricular (RV) leads are the main risk factors causing TR [25]. The lead is attached to the TV or right ventricle wall by a strong connective scar tissue [26]. Moreover, a higher proportion of those suffering from acute TR had longer post-extraction hospital stays and experienced more frequent TV apparatus damage [25].

Patients with extensive TV damage had much lower rates of full clinical success and full procedural success, but there was no connection between the decline in TV function and long-term survival [26]. According to Polewczyk et al., 90.31% who underwent TLE did not develop TR [26]. However, 9.70% of the patients experienced insignificant worsening degrees of TR. Only 2.54% of the patients developed notable decreases in TV function [26]. Similarly, 24 out of 208 patients (11.5%) in study number 25 experienced TR after TLE [25]. Only one patient needed urgent TV replacement surgery for new-onset TR caused by the avulsion of the TV septal leaflet [25]. Diuretics were used to treat the remaining 23 patients [25]. 

According to Tułecki et al., 115 (7.67%) patients experienced modest TLE-related complications [19]. TV damage, which progressed by two or three degrees in 43 patients (2.91%), was the most frequent complication [19]. One degree of deterioration was not considered a modest consequence because it could be quite subtle and lead to fluid accumulation [19]. 

Vascular Complications 

The most terrifying complications of TLE include cardiac avulsion or tear (CA/T) with tamponade and vascular avulsion or tear (VA/T) due to superior vena cava (SVC) laceration [33]. The most frequent sites of cardiac laceration/vascular wall tear (CVWT) are the connection of the SVC to the right atrium (RA) [19]. Among the 58 patients, 49 (84.5%) had a CVWT (30 cardiac and 19 vascular) [33]. The success rates for treating cardiac avulsion with tamponade and vascular tears were 83.33% and 85.37%, respectively, while the mortality rates for patients with CA/T and VA/T were 20% and 31.6%, respectively [33]. Pericardiocentesis, sternotomy, or thoracotomy may be necessary in certain cases when cardiac tamponade complicates CA/T [33]. 

Numerous risk factors, such as implanting the device on the right side, maintaining leads in place for longer than 10 years, removing three or more leads, and using the femoral approach during the procedure, may increase the likelihood of VA/T [33]. Contrarily, patients using anticoagulants demonstrated less occlusive consequences after laser lead extraction, as the laser can cause vascular abrasions, which can enhance the formation of clots and occlusion [34]. Using anticoagulants makes it easier and more successful for cardiologists to implant new CIEDs [34].

Pseudoaneurysm of the SVC or inferior vena cava (IVC) is one of the most uncommon vascular complications of TLE [29]. Hayashi et al. revealed two cases of this condition, and possible explanations include the pressure differential between the venous and arterial systems or adhesions between the pleura and mediastinal tissues [29]. Physicians recommend conservative treatment for this condition. However, if it ruptures, it could cause sudden death, so a computed tomography (CT) scan is advised to check for any SVC damage and determine whether it has expanded [29].

Only a single case of disseminated intravascular coagulopathy (DIC) being a complication of CIED lead extraction has been documented [30]. It has been noted that the primary causes of DIC, in this case, include surgery itself, infection, and especially sepsis [30]. An inflammatory response to infection and sepsis known as DIC causes the immune system to become activated, producing cytokines and chemokines and activating the coagulation cascade, which ultimately results in organ failure [30]. In the case of uncontrolled bleeding post CIED extraction, you should suspect DIC especially when multiple sites are oozing blood [30]. Early diagnosis and treatment with packed red blood cells, fresh frozen plasma, platelets, and cryoprecipitate will decrease mortality and morbidity [30].

Ghost Appearance in Echo 

The primary pathophysiology of ghost development is the invasion of lead capsules by fibrous tissue and blood vessel migration [31]. It is difficult for leads to move because of this interaction between heart tissues and leads, which also makes thrombus formation possible [31,35,36]. ​​The most reliable indicator of ghosts is how much the connective tissue surrounding the lead has grown and developed before TLE [27]. Transesophageal echocardiogram (TEE) proved to be an appropriate tool for imaging scar tissue encompassing the leads [27].

The newly removed masses that may be seen by TEE before or after extraction are known as stable ghosts [27]. They are tubular and echogenic, with one end connected to any part of the heart and the other floating freely [27,37]. However, flying ghosts are formed when the leads are released and the surrounding fibrous tissues are severed. The masses either stay attached to the cardiovascular structures as the dilating sheath is moved downward or they become free after many circular rotations [27].

The study's most important finding is that the existence of the two types of ghost had no impact on how long people survived after TLE [27]. In addition, it has been observed that freed ghosts migrate to the pulmonary vascular bed and disappear in around 15% of the cases [27]. However, stable ghosts remain stuck to the CVS wall in about 30% of patients [27].

The current findings demonstrate that the number of leads and implant dwelling time were the main predictors of ghost formation after TLE if the cause of TLE was not the infection [27]. However, a higher NYHA class, the presence of any renal failure, and resynchronization therapy before TLE were all linked with shorter survival in the infected group [27]. Moreover, patients with higher left ventricular ejection fraction (LVEF) and female patients seemed to have better outcomes [27].

Old Age

The number of older people with CIED is steadily increasing as the world's population ages [14]. Due to their potentially worse overall health, higher rates of concurrent disorders, and added challenges during surgery, TLE safety is a significant issue for discussion [14]. According to Zhou et al., the most common indication for TLE Is device-related infection (93.5%) in octogenarians [14]. Similarly, study number 21 showed that infection-related devices, whether pocket infection or systemic infection, had an indication for TLE in 77.5% of the study population [21]. By contrast, Zabak et al. demonstrated that infection indications of TLE represent 33.3% of the octogenarian population [24]. In study number 24, even though octogenarians have lower ejection fraction, hemoglobin levels, and kidney function along with associated comorbidities, such as CAD, atrial fibrillation, cerebrovascular diseases, and diabetes mellitus [24]. The proportions (98.9% and 96.7%) of the patients in study number 14 showed clinical and procedural success, respectively, which is nearly identical to that of the non-octogenarian group (Table 9).

Major complications included cardiac tamponade requiring pericardiocentesis and right atrial perforation with pericardial effusion, which was managed by emergent sternotomy [14,21]. Moreover, hemothorax and pocket infection were reported as minor complications [14,21]. Meanwhile, study number 24 identified no major complications, which illustrate that TLE is a safe procedure in the elderly regardless of all comorbidities [24]. However, we noticed that 30-day all-cause mortality was greater in octogenarians than in the younger population in our study (study number 8). Proportions of 5.6% and 1.9% were reported in the octogenarians and the younger group, respectively [24]. LVEF decline, anemia, the number of leads extracted in the procedure, and device-related infective endocarditis are the reported factors that have an impact on the 30-day all-cause death rate [24].

**Table 9:** Prevalence of indications, success, and complications rates of TLE in the elderly

	Year of publication	Number of patients with leads extracted	Number of leads extracted	Number of patients with infection indication / %	Number of patients with non-infection indication / %	Clinical success rate	Procedure success rate	Major complication number / (%)	Minor complication number / (%)
Zhou et al. [14]	2021	Non-octogenarian: 922 patients; octogenarian: 184 patients	Octogenarian:378 leads; non-octogenarian: 2004 leads	Non-octogenarian: 810 (87.8%); octogenarian: 172 (93.5%)	Non-octogenarian: 112 (12.2%); octogenarian: 12 (6.5%)	Non-octogenarian: 909 (98.6%); octogenarian: 182 (98.9%)	Non-octogenarian: 879 (95.3%); octogenarian: 178 (96.7%)	Non-octogenarian: 10 (1.1%); octogenarian: 2 (1.1%)	Non-octogenarian: 18 (1.9%); octogenarian: 3 (1.6%)
Zabek et al. [24]	2020	Non-octogenarian: 577 patients; octogenarian: 90 patients		Non-octogenarian: 99 (17.1%); octogenarian: 30 (33.3%)	Non-octogenarian: 478 (82.9%); octogenarian: 60 (66.7%)		Non-octogenarian: 554 (96%); octogenarian: 88 (97.8%)	Non-octogenarian: 9 (1.6%); octogenarian: 0	Non-octogenarian: 9 (1.6%); octogenarian: 2 (2.2%)
Burger et al. [21]	2021	71	152	32 (45.1%)	39 (54.9%)	70 (98.6%)	66 (92.9%)	1 (1.4%)	2 (2.8%)

Old Leads 

There is a higher possibility of extraction failure, incomplete procedural success, and generally more complications, especially in extremely old leads. However, using several extraction tools could boost the success rate of the technique [28]. Using mechanical rotational sheaths or snares was not associated with a higher incidence of serious complications [28]. According to Ząbek et al., the procedural success rate was 95.6% for leads implanted over 10 years (Group A) and 99.6% for leads implanted within 10 years (Group B) [22]. Similarly, in research number 23, the clinical success rate for the older lead was lower than for the younger ones (89.81% vs. 96.60%) (Table 10). 

Major complications, including SVC perforation, laceration of the RA at the level of the SVC, perforation of the right atrial appendage, pericardial effusion requiring a pigtail catheter, and TV damage indicating surgery, were reported [28]. A significant increase compared to earlier trials (0.9-2.5%), this research had a higher major complication rate of 3.3% [28]. 

In study number 22, infection-related problems formed 20.6% of the patients [22]. The probability of infection increases with the length of the leads' dwell time. In addition, the procedure itself has shown to be riskier over time, especially when there is evidence of an infection, due to a rise in lead adhesions and fibrous tissue [23]. According to Segreti et al., infection was the primary reason to perform TLE in 78.81% of cases, compared to non-infection causes in 21.56% of cases [23]. This guarantees the connection between lead aging and infection and how it may impact results.

**Table 10:** Comparing patients with leads implanted over 10 years (Group A) to leads within 10 years (Group B)

	Year of publication	Number of patients with leads extracted	Number of leads extracted	Number of patients with infection indication / (%)	Number of patients with non-infection indication / (%)	Clinical success rate	Procedure success rate	Major complication number/ (%)	Minor complication number / (%)
Ząbek et al. [22]	2023	Patient with old leads (A): 46; patient with younger leads (B): 272	Group A: 66; Group B: 439	Group A: 9 (20.6%); Group B: 92 (33.8%)	Group A: 37 (80.4%); Group B: 180 (66.2%)		Group A: 44 (95.6%); Group B: 271 (99.6%)	Group A: 3 (6.5%); Group B: 4 (1.5%)	Group A: 1 (2.2%), Group B: 5 (1.8%)
Segreti et al. [23]	2019	Patient with old leads (A): 422; patient with younger leads (B): 3,088		Group A: 331 (78.81%); Group B: 1,534 (49.82%)	Group A: 91 (21.56%); Group B: 1,554 (50.32%)	Group A: 379 (89.81%); Group B: 2,983 (96.60%)		Group A: 23 (5.45%); Group B: 72 (2.33%)	Group A: 35 (8.29%); Group B: 139 (4.50%)
Pecha et al. [28]	2021	154 patients with old leads	362	98	56	149 (96.8%)	141 (91.6%)	5 (3.3%)	2 (1.3%)

Limitation

Some of the studies that were excluded during screening should have been discussed, but the full texts for these papers were not readily available. In addition, long-term prognosis and complications after years of TLE were not broadly covered in the studies. Furthermore, details of the treatment and interventions taken to treat the complications need to be addressed.

## Conclusions

TLE is the best option for treating leads with an infection or malfunction. It is crucial to list all the common and rare complications that might come up during the procedure and the postoperative complications to get ready for such situations. TLE is a safe procedure that has a high chance of procedural and clinical success in both young and elderly patients. Even if cardiac and vascular tear, as well as TR, are the most common complications during TLE, their prevalence compared to success rates remains too low. In addition, as time passes after implantation, extraction complications become more likely due to fibrotic adhesions between the leads with the heart and vascular tissues. Future studies should evaluate and assess complications and mortality rates after the surgery has been performed for an extended period of years.
